# Impact of sepsis on circulating exosomes: pathophysiological mechanisms, biomarker potential, and therapeutic applications

**DOI:** 10.1097/JS9.0000000000003129

**Published:** 2025-08-08

**Authors:** Shao-Chun Wu, Cheng-Shyuan Rau, Ching-Hua Hsieh

**Affiliations:** a Department of Anesthesiology, Kaohsiung Chang Gung Memorial Hospital and Chang Gung University College of Medicine, Taiwan; b Graduate Institute of Clinical Medical Sciences, College of Medicine, Chang Gung University, Taoyuan, Taiwan.; c Department of Neurosurgery, Kaohsiung Chang Gung Memorial Hospital and Chang Gung University College of Medicine, Taiwan; d Department of Plastic Surgery, Kaohsiung Chang Gung Memorial Hospital and Chang Gung University College of Medicine, Taiwan

**Keywords:** biomarkers, exosomes, extracellular vesicles, inflammation, sepsis

## Abstract

This comprehensive review examines the complex interplay between sepsis and circulating exosomes. Sepsis, characterized by dysregulated host response to infection leading to life-threatening organ dysfunction, remains a significant global health challenge with high mortality rates. Exosomes, small extracellular vesicles (30-150 nm) released by most cell types, play crucial roles in intercellular communication by transferring bioactive molecules. During sepsis, both exosome quantity and cargo composition undergo significant alterations, reflecting the host’s pathophysiological state. The review explores how sepsis-induced inflammation influences exosome biogenesis and content, including proteins, microRNAs, and other non-coding RNAs. These modified exosomes can propagate inflammatory signals throughout the body while also participating in immunosuppressive mechanisms characteristic of later sepsis stages. The potential of exosomes as diagnostic and prognostic biomarkers is highlighted, with specific exosomal components correlating with disease severity and organ dysfunction. Additionally, emerging therapeutic strategies targeting exosomes or utilizing them as delivery vehicles for anti-inflammatory agents are discussed. By consolidating recent findings, this review underscores the significance of exosome research in advancing our understanding of sepsis pathophysiology and developing novel interventions for this complex syndrome.

## Introduction

Sepsis is a severe condition characterized by life-threatening organ dysfunction triggered by a dysregulated host response to infection^[1]^. It remains a leading cause of morbidity and mortality worldwide, with an estimated 50 million cases and 11 million sepsis-related deaths annually^[2]^. Despite advances in supportive care, the heterogeneity of sepsis and lack of specific diagnostic tests pose major challenges in timely diagnosis and effective treatment^[2,3]^. Early biomarkers and targeted therapies are urgently needed to improve patient outcomes in sepsis.

**HIGHLIGHTS**

- Sepsis alters exosome quantity and cargo composition, reflecting host inflammatory state
- Exosomes mediate pro-inflammatory signals in early sepsis and immunosuppression in later stages
- Exosomal proteins, microRNAs, and non-coding RNAs serve as potential sepsis biomarkers
- Exosome profiles correlate with sepsis complications in multiple organ systems
- Therapeutic strategies include inhibiting harmful exosomes and utilizing beneficial ones

Exosomes are small (30–150 nm) extracellular vesicles of endosomal origin released by virtually all cell types^[1,2]^. They carry a complex cargo of proteins, lipids, and nucleic acids (mRNAs, microRNAs, long noncoding RNAs, etc.) reflective of their cell of origin, and can transfer this cargo to target cells, modulating recipient cell functions^[1,2]^. In normal physiology, exosomes mediate intercellular communication and immune regulation, but in pathological states like infection and inflammation, exosome production and content can be profoundly altered^[4]^. There is growing evidence that exosomes play a significant role in sepsis pathogenesis by disseminating inflammatory signals and coordinating immune responses across organs^[2,3]^. Because exosomal cargo is protected by a lipid bilayer, these vesicles are stable in circulation and can serve as robust biomarkers mirroring the host’s pathophysiological state^[2,5]^.

This review provides an overview of the impact of sepsis on circulating exosomes, with a focus on how the septic inflammatory milieu influences exosome biogenesis, cargo, and functional effects. We conducted a comprehensive literature search in PubMed (MEDLINE), Scopus, and Web of Science for articles published from 2013 to 2025, using combinations of search terms related to sepsis and exosomes (including “sepsis,” “exosomes,” “extracellular vesicles,” “biomarkers,” and “therapy”). We restricted the search to peer-reviewed, English-language publications. We included studies that presented original data on sepsis and circulating exosomes (extracellular vesicles) in humans, animals, or relevant in vitro models, and excluded non-English publications, review articles lacking original data, and studies not specifically addressing sepsis-related exosomes. Given the narrative and non-systematic nature of this review, we did not use a PRISMA flow diagram for study selection. Likewise, we did not apply formal quality appraisal tools (e.g., SANRA for narrative reviews or AMSTAR 2 for systematic reviews), as our aim was to provide a broad qualitative reviewing of the literature rather than a systematic, quantitative evaluation of study quality. This narrative review provides a novel integrated analysis of circulating exosomes in sepsis, bridging diagnostic and therapeutic perspectives that have rarely been addressed together in previous reviews. It synthesizes emerging evidence establishing exosomes as diagnostic and prognostic biomarkers correlated with disease severity and organ injury. Furthermore, it underscores the paradoxical dual roles these vesicles play in sepsis pathophysiology – capable of propagating systemic inflammatory injury while also mediating tissue repair. This multifaceted approach distinguishes our review from prior literature and provides a timely, holistic perspective on the implications of exosomes in sepsis. In this study, we first outline the immunopathology of sepsis, then examine observed changes in exosome quantity and composition during sepsis and their functional consequences. Furthermore, we discuss clinical implications, including the potential of exosomes as diagnostic/prognostic biomarkers and emerging therapeutic strategies that target or harness exosomes in sepsis. By consolidating recent findings (primarily from the last 5 years), we aim to highlight the significance of exosome research in understanding sepsis pathophysiology and in developing novel interventions for this complex syndrome.

## Pathophysiology of sepsis

Sepsis involves a dysregulated immune response with an initial hyperinflammatory phase followed by immunosuppression^[6]^. The early phase features excessive pro-inflammatory mediator release – a “cytokine storm” – causing widespread tissue inflammation, endothelial dysfunction, and coagulopathy^[2,6]^. This hyperinflammation is triggered by pathogen-associated molecular patterns and amplified by damage-associated molecular patterns (DAMPs) from injured cells^[1,2]^. Activated immune cells release large quantities of cytokines such as tumor necrosis factor alpha (TNF-α), interleukin-1β (IL-1β), and IL-6, many packaged into exosomes^[2,4]^. Septic plasma exosomes carry elevated inflammatory cytokines compared to healthy individuals, propagating inflammation to distant sites and activating immune cells^[1,2]^. Gao *et al* (2019) showed septic mouse exosomes stimulate T lymphocyte proliferation and chemotaxis^[1,7]^, contributing to systemic inflammatory response syndrome.

Concurrently, sepsis triggers immunosuppressive mechanisms including accelerated lymphocyte apoptosis and impaired antigen presentation, leading to immune “paralysis”^[1,6]^. Exosomes participate in this immunosuppressive switch by carrying immune checkpoint molecules like programmed death-ligand 1 and 2 (PD-L1/PD-L2)^[8]^. Kawamoto *et al* (2019) found exosomal PD-L2 and β2-integrin significantly increased in septic patients, potentially contributing to T-cell inhibition^[8]^. Sepsis-derived exosomes also carry immunosuppressive microRNAs like miR-127 and miR-125b, implicated in monocyte deactivation and T-cell exhaustion^[4]^.

Sepsis also disturbs coagulation and endothelial integrity. Activated monocytes release tissue factor-positive exosomes that promote coagulation, correlating with disseminated intravascular coagulation (DIC)^[2,9]^. However, granulocyte-derived microvesicles expressing plasminogen activators enhance fibrinolysis and improve outcomes^[2,3]^, while neutrophil-derived exosomes enriched with superoxide dismutase 2 mitigate coagulopathy by reducing oxidative endothelial injury^[3]^. This demonstrates exosomes’ context-dependent effects – some promote clotting while others facilitate resolution.

Sepsis-induced organ injury is influenced by exosomal signaling from both host and pathogen sources. Bacterial outer membrane vesicles carrying endotoxin trigger inflammatory cascades and distant organ damage^[2,10]^. Host cells counter this through defensive exosomes – Keller *et al* (2020) showed human cells release ADAM10-carrying exosomes that neutralize bacterial toxins, improving survival in infected mice^[11]^. However, sepsis-associated exosomes also cause direct organ injury, inducing vascular apoptosis and myocardial dysfunction^[12,13]^. Specific microRNAs (miR-126-5p, miR-146a, miR-21) alter endothelial permeability and organ function, linking systemic inflammation with multi-organ failure^[4,13]^. Thus, exosomes in sepsis both exacerbate inflammation and provide protective feedback, making them important biomarkers and therapeutic targets.

## Impact of sepsis on exosomes

The effect of sepsis on circulating exosomes is explored here, with current examples of studies on exosome changes during sepsis given in Table 1 and pathophysiological changes in sepsis mediated by exosomes listed in Table 2.

**Table 1:** Studies on changes in exosome quantity and quality during sepsis. This table summarizes select studies that have characterized alterations in circulating exosome levels and cargo in sepsis, highlighting their key findings

Study (year)	Subjects/model	Exosome changes observed	Key findings	reference
Gao *et al*, 2019	Mouse sepsis (CLP)	↑ Exosome count in septic serum; exosomes carry cytokines (IL-6, TNF-α) and chemokines	Exosomal cytokines activated T-cells via TLR4, promoting T cell proliferation and chemotaxis (hyperinflammation)	^[7]^
Deng *et al*, 2019	Human patients (septic ICU)	Plasma exosomal miR-7-5p ↑ in sepsis	Exosomal has-miR-7-5p delivered to T cells; downregulated pro-apoptotic Bad, reduced T-cell apoptosis (potentially mitigating lymphopenia)	^[14]^
Dakhlallah *et al*, 2019	Human patients (septic shock)	Circulating EV mRNA profile change: ↑ DNMT1/3A/3B mRNA in EVs	EV cargo revealed induction of DNA methyltransferases in sepsis; proposed as molecular predictors of septic shock outcomes	^[15]^
Morris *et al*, 2020	Human patients (ED sepsis)	Proteomic changes: ↑ acute-phase proteins (SAA, CRP) and Igs in exosomes vs. controls	Exosomes from septic patients showed enriched inflammatory and complement proteins, reflecting early acute-phase response. Differentiated sepsis vs. healthy profiles	^[16]^
Real *et al*, 2018	Human patients (septic shock)	Distinct exosomal miRNA signature (inflammation & cell cycle-related miRNAs)	Septic shock exosomes conveyed miRNAs (e.g., miR-223, miR-146b) affecting inflammatory and cell-cycle pathways; these exosomes induced endothelial apoptosis and myocardial dysfunction in vitro	^[12]^
Xu *et al*, 2018	Human patients (sepsis)	Exosomal proteome dynamics during sepsis progression	Early sepsis: ↑ complement and coagulation factors in exosomes; late sepsis: shift to proteins related to immune suppression. Exosomal protein changes tracked disease course, suggesting use in monitoring.	^[17]^
Kawamoto *et al*, 2019	Human (sepsis vs. SIRS)	↑ Exosome surface PD-L2 and β2-integrin in sepsis; exo PD-L1 unchanged (soluble PD-L1 ↑)	EV phenotyping differentiated sepsis from sterile inflammation. Exosomal PD-L2 correlated with immunosuppression severity; total circulating PD-L1 (exo + soluble) higher in sepsis.	^[18]^
Tian *et al*, 2021	Human patients (sepsis)	↑ Exosomal circRNAs (circ_104484, circ_104670) in sepsis vs. controls	Identified two upregulated exosome-derived circRNAs in sepsis; ROC analysis showed high diagnostic accuracy for sepsis. These circRNAs proposed as novel stable biomarkers for sepsis.	^[19]^

**Table 2:** Pathophysiological changes in sepsis mediated by exosomes. This table highlights how exosomes contribute to various pathological or protective processes in sepsis, including inflammation amplification, immune suppression, coagulopathy, and organ damage

Exosomal source/content	Pathophysiological role in sepsis	Mechanism/effect	Outcome	Reference
Monocyte-derived TF + exosomes	Coagulation activation (DIC)	Exosomes expose tissue factor -> trigger extrinsic coagulation pathway. Increased procoagulant activity correlates with sepsis severity	Microthrombi formation; DIC development	^[9]^
Neutrophil exosomes with α-2-macroglobulin	Anti-bacterial, pro-resolving function	α-2-Macroglobulin on exosomes binds and neutralizes bacterial proteases/toxins, aiding clearance	Reduced inflammation; improved bacterial clearance (enhanced survival in sepsis)	^[20]^
Macrophage exosomal miR-155	Inflammation amplification	Exosomal transfer of miR-155 to naive macrophages -> suppresses SOCS1 & SHIP1, leading to NF-κB hyperactivation	Macrophage M1 polarization; ↑ TNF-α, IL-6 production (cytokine storm exacerbation)	^[21]^
T-cell exosomal PD-L1/PD-L2	Immune suppression (late sepsis)	Exosomal PD-L2 (↑ in sepsis) engages PD-1 on T cells -> T-cell inhibition. Exosomal PD-L1 adds to soluble PD-L1 pool, contributing to T-cell exhaustion	Lymphocyte anergy/apoptosis; impaired pathogen clearance (susceptibility to secondary infection)	^[8]^
Platelet-derived exosomes (sepsis)	Myocardial dysfunction, vasodilation	Contain NADPH oxidase components -> generate nitric oxide and ROS in heart endothelium. Induce cardiomyocyte depression and vascular leakage	Septic cardiomyopathy; hypotension	^[22]^
Endothelium-derived exosomes with HSPs/DAMPs	Endothelial activation & permeability	Carry HSP70, HMGB1, etc. -> activate endothelial TLR4 and inflammasome pathways in distant vasculature	Capillary leak, tissue edema (organ dysfunction)	^[22]^
Bacterial outer membrane vesicles (OMVs)	Exogenous inflammatory triggers	Gram-negative OMVs deliver LPS into host cells -> caspase-11 activation and cytokine release. Gram-positive EVs carry toxins -> direct host cell injury	Exacerbation of systemic inflammation; direct cytotoxic effects	^[10]^
Exosomes carrying ADAM10 (decoy exo)	Host defense against toxins	ATG16L1-dependent exosome release with ADAM10 receptors that sequester bacterial α-toxin (*S. aureus*)	Neutralization of pore-forming toxins; protects host cells from lysis (increased survival in infection)	^[11]^
Exosomal miR-1262 (from septic patient)	Metabolic and cell survival dysregulation	Uptake by cardiomyocytes -> silences SLC2A1 (GLUT1) and impairs glycolysis; also promotes apoptosis in heart cells	Cardiac energy failure and apoptosis; contributes to septic cardiomyopathy	^[13]^
Exosomal let-7 family miRNAs	Anti-inflammatory modulation (resolution phase)	Transfer to macrophages -> suppress HMGB1 and IL-6 expression via post-transcriptional regulation	Reduced cytokine output; promotes resolution of inflammation	^[23]^
Exosomal NEAT1 lncRNA (from neurons/glia)	Pathogenesis of sepsis-associated encephalopathy (SAE)	Exosomal NEAT1 (↑ in sepsis) taken up by microglia -> promotes ferroptosis by sponging miR-9-5p, leading to neuronal iron overload	Exacerbation of brain injury, cognitive dysfunction in SAE	^[24]^
Exosomal lncRNA-p21 (from MSCs)	Protective in lung injury	MSC exosomes deliver lncRNA-p21 to lung epithelium -> lncRNA-p21 sponges miR-181, relieving inhibition on pro-survival pathways	Decreased alveolar cell injury; attenuated LPS-induced acute lung injury	^[25]^

*Changes in Exosome Quantity and Composition in Sepsis*: Circulating exosomes are significantly elevated in sepsis patients compared to healthy controls, with concentrations correlating with disease severity^[2]^. Septic plasma exosomes exhibit markedly different molecular profiles from those of healthy individuals^[4]^. Proteomic analyses reveal sepsis exosomes are enriched in acute-phase proteins and inflammatory mediators. Morris *et al* (2020) found upregulated acute-phase reactants like C-reactive protein and serum amyloid A in septic patients’ exosomes^[16]^. Xu *et al* (2018) showed exosomal proteome changes during sepsis include increasing complement factors and coagulation proteins, contributing to ongoing inflammation and coagulopathy^[17]^.

Sepsis also alters exosomal RNA cargo. Circulating exosomal mRNAs related to oxidative stress defenses (peroxiredoxins, superoxide dismutase) are elevated, possibly reflecting compensatory responses to oxidative injury^[2]^. MicroRNA profiling identifies sepsis-specific signatures: multiple microRNAs (miR-146a, miR-155, miR-125b) that modulate inflammation and immune function are differentially expressed in septic versus control exosomes^[2,4]^. Real *et al* (2018) found septic shock patients’ exosomes carried distinct miRNAs related to inflammation and cell cycle regulation, suggesting broad reprogramming of exosomal microRNA content^[12]^. Non-coding RNAs are similarly affected – exosomal NEAT1 (a lncRNA) is increased and linked to sepsis-associated encephalopathy^[24]^, while circRNA_104484/104670 show diagnostic potential^[22]^.

The balance of pro- versus anti-inflammatory exosomal components shifts dynamically during sepsis progression. In the early hyperinflammatory phase, exosomes are loaded with pro-inflammatory cytokines and DAMPs. Septic exosomes often contain High Mobility Group Box 1 (HMGB1) and heat shock proteins that perpetuate inflammation^[4]^. Elevated HMGB1 in sepsis exosomes induces pyroptotic cell death and further cytokine release in target cells^[4]^. Exosomal IL-1β, IL-6, and IL-10 are also increased; certain cytokines (IL-1β, IL-12, IL-17) peak earlier within exosomes than in plasma, suggesting exosomes serve as early cytokine reservoirs^[2]^.

As sepsis progresses to the immunosuppressive phase, exosomes increasingly carry anti-inflammatory cargo. Exosomal PD-L2 is elevated and dampens T-cell responses^[6]^. Anti-inflammatory microRNAs like miR-146a (NF-κB negative regulator) and miR-21 are upregulated, contributing to monocyte deactivation^[4]^. Dakhlallah *et al* (2019) found increased DNA methyltransferase mRNA in the extracellular vesicles of septic shock patients, suggesting exosomes deliver epigenetic regulators that shift immune phenotypes toward tolerance^[21]^. In summary, sepsis drives dynamic alterations in both exosome quantity and molecular composition, creating a molecular “fingerprint” of the septic state. The cargo initially propagates inflammation but gradually incorporates factors promoting immune suppression and tissue repair, reflecting the biphasic nature of sepsis pathophysiology.

*Functional Alterations of Exosomes During Sepsis* – Sepsis-derived exosomes exert potent biological effects that recapitulate the host’s dysregulated immune responses. Exosomes from septic patients induce apoptosis in endothelial cells and cardiac myocytes, contributing to vascular leakage and septic cardiomyopathy^[12–14]^. In one study, serum exosomes from sepsis patients markedly reduced glycolytic activity and induced apoptosis in cultured myocardial cells through exosomal transfer of hsa-miR-1262, which downregulates the glucose transporter SLC2A1^[13]^. This mechanism illustrates how exosomal microRNAs cause metabolic reprogramming and cell death in distant organs, impairing cardiac energy metabolism and function. Similarly, miR-155-enriched exosomes from endotoxin-stimulated macrophages promote inflammatory activation by triggering M1 polarization and increasing TNF-α and IL-6 production in recipient macrophages, thereby amplifying inflammation^[19]^.

In T lymphocytes, sepsis exosomes can have divergent effects: Deng *et al* (2019) found that exosomes from septic plasma delivered miR-7-5p to T cells, which suppressed the pro-apoptotic protein Bad and protected T cells from apoptosis^[1,25]^, potentially contributing to the expansion of certain T-cell subsets and influencing the balance of T helper or regulatory T cells. Conversely, prolonged sepsis leads to exosomes that inhibit T-cell proliferation and cytokine production through PD-L1 or transforming growth factor–β (TGF-β) cargo, reinforcing T-cell exhaustion^[6]^.

Exosomes mediate organ-specific injury in sepsis. In the lungs, they contribute to acute respiratory distress syndrome (ARDS) – neutrophil-derived exosomes prime lung macrophages and endothelial cells to produce pro-inflammatory mediators, worsening pulmonary inflammation^[2]^. However, some exosomes are protective: bronchial epithelial cell-derived exosomes containing let-7 family microRNAs reduce macrophage activation and limit lung injury in experimental models^[4]^. In kidneys, sepsis-induced acute kidney injury involves circulating exosomes delivering injury signals to renal tubular cells. Exosomal miR-93-5p from endothelial progenitor cells normally suppresses kidney inflammatory pathways; studies show boosting miR-93-5p in exosomes protected mice from acute kidney injury (AKI) by targeting KDM6B and reducing NF-κB-driven inflammation in renal cells^[3]^. If sepsis impairs the release of such protective exosomes, or if circulating pathogenic exosomes overwhelm them, the result is enhanced renal inflammation and cell death.

Importantly, many exosomal contents in sepsis have biomarker potential. Since exosomes encapsulate molecules from parent cells, specific proteins or RNAs in circulating exosomes indicate activation or damage of certain cell types. Elevated exosomal aminopeptidase N (CD13) associates with sepsis-induced acute lung injury; macrophage-derived CD13-rich exosomes induce necroptosis of lung epithelial cells, linking exosome cargo with lung injury mechanisms^[5]^. Urinary exosomal NHE3 (sodium/hydrogen exchanger 3) significantly increases in sepsis patients developing AKI, carrying early signals of kidney tubular stress and serving as a predictive biomarker for incipient AKI^[5]^. Exosomal DNA and mitochondrial DNA are also valuable – elevated mitochondrial DNA in exosomes associates with poor prognosis, reflecting mitochondrial DAMP release^[5]^. Thus, exosomes provide a rich source of biomarkers whose cargo profiles reveal ongoing pathophysiological processes in sepsis not evident from free-circulating molecules alone^[5]^.

*Specific Biomarkers Associated with Sepsis-Related Exosome Changes* – Several exosomal components serve as sepsis indicators. Among protein biomarkers, exosomal HMGB1 is elevated in sepsis patients and correlates with disease severity and outcomes, potentially predicting septic shock or death more effectively than total HMGB1 levels^[4]^. Extracellular cold-inducible RNA-binding protein (eCIRP), another DAMP released in exosomes during sepsis, has been proposed as a marker for both sepsis and organ injury^[5]^.

Recent studies have identified multiple exosomal protein biomarkers for sepsis complications. Exosomal Galectin-9 is significantly upregulated in sepsis and correlates with worse organ dysfunction scores^[5]^. In sepsis with acute respiratory distress, exosomal CD13/APN indicates macrophage activation and drives inflammatory cell death in the lung, while exosomal histones promote coagulation and endothelial damage^[4,5]^. For septic encephalopathy, exosomal AQP4 (astrocyte water channel protein) is elevated in patients with brain dysfunction^[5]^. In septic shock, increased exosomal TREM-2 (Triggering Receptor on Myeloid cells-2) reflects monocyte/macrophage activation and associates with shock severity^[5]^. Conversely, some proteins decrease – exosomal SPTLC3, an enzyme in sphingolipid metabolism, is downregulated and negatively correlates with disease progression^[4]^. This diversity of exosomal protein changes provides multiple candidates for biomarker panels.

## MicroRNA biomarkers

MicroRNAs are attractive sepsis biomarkers due to their stability within exosomes and ease of profiling. Exosomal miR-122, a liver-enriched microRNA, is significantly elevated in sepsis and correlates with short-term mortality, serving as both a prognostic indicator and marker of hepatic dysfunction^[4]^. MiR-146a, upregulated in septic exosomes as a negative feedback inflammation regulator, signals compensatory anti-inflammatory responses and has been linked to better outcomes^[4]^. Conversely, pro-inflammatory exosomal miR-155 rises in early sepsis, marking hyperinflammation and associating with increased monocyte activation and worse inflammatory injury^[1,4,19]^. Hermann *et al* (2020) reported that a panel of three exosomal microRNAs – miR-21, miR-221, and miR-15a – distinguishes sepsis from non-septic critical illness, highlighting their diagnostic potential^[2]^.

For organ-specific injury, exosomal microRNAs serve as both mediators and markers. Neutrophil-derived exosomal miR-30d-5p induces macrophage pyroptosis and lung injury, with high levels linked to ARDS development^[33]^. In contrast, exosomal miR-93-5p from endothelial progenitor cells shows protective effects in acute kidney injury, predicting renal recovery when present at high levels^[3,5]^. It is likely that a combination of several exosomal miRNAs will yield the most robust diagnostic or prognostic signatures for sepsis. Ongoing research is defining exosomal miRNA panels that can diagnose sepsis earlier or stratify patients by likelihood of organ failure^[2,5]^.

Long noncoding RNAs and circRNAs in exosomes are emerging as important sepsis biomarkers. Exosomal NEAT1 is consistently elevated in sepsis patients, particularly those with encephalopathy, correlating with disease severity and brain injury markers^[4,24]^. TapSAKI (sepsis-associated kidney injury lncRNA) is upregulated in exosomes during septic AKI and promotes inflammatory kidney injury, serving as both a biomarker and potential therapeutic target^[4]^. Conversely, exosomal TUG1 is downregulated in sepsis, with low levels associated with worse AKI outcomes^[4]^. These findings highlight how different exosomal lncRNAs can indicate specific organ injuries and disease severity in sepsis.

For circRNAs, Tian *et al* (2021) identified circRNA_104484 and circRNA_104670 as significantly elevated in serum exosomes of sepsis patients, demonstrating good diagnostic accuracy (AUC > 0.85)^[22]^. These stable circular RNAs represent excellent blood biomarkers. Another circRNA, circFADS2, decreases in sepsis exosomes and may play a protective role in lung cells^[5]^. As research advances, the expanding repertoire of exosomal biomarkers – including proteins, miRNAs, lncRNAs, and circRNAs – holds promise for comprehensive diagnostic panels that could detect sepsis and predict complications earlier and more reliably than current methods through simple blood draws.

## Clinical implications

*Exosomes as Biomarkers for Sepsis Diagnosis and Monitoring* – The profound alterations in exosomal content during sepsis make them promising clinical biomarkers. Traditional sepsis biomarkers like procalcitonin and C-reactive protein have limitations in sensitivity and specificity^[5]^, while exosomes offer advantages: they exist in all body fluids, their membrane protects cargo from degradation, and their molecular contents reflect ongoing pathophysiological processes in real time^[2,5]^. Exosomal biomarkers enable earlier sepsis diagnosis, differentiation from non-infectious systemic inflammatory response syndrome, and organ-specific injury monitoring. For instance, circulating exosomal microRNA profiles distinguished sepsis from pneumonia alone in pneumonia patients^[2]^. Cell-specific exosomes provide organ-targeted biomarkers – endothelial-derived exosomes carrying HSPA12B associated with septic cardiomyopathy severity, while platelet-derived exosomes linked to myocardial depression^[2,5,14]^. Yuan *et al* (2024) demonstrated that certain exosomal proteins outperformed conventional markers in identifying sepsis-induced organ damage, noting that exosomal MT-ND6 predicted cardiac dysfunction earlier than echocardiographic changes^[5]^.

A comprehensive plasma exosome panel for intensive care could include exosomal IL-6 and IL-10 for inflammation, PD-L1 or miR-146a for immunosuppression status, miR-122 for liver injury, NHE3 or miR-21-5p for AKI, and bacterial vesicle components to confirm infection^[3,5]^. While such comprehensive assays aren’t yet clinical reality, newer technologies like microfluidic chips, immunoaffinity capture, and nanoparticle tracking are making exosome detection more feasible in clinical labs^[5]^. Some assays now measure exosomal microRNAs directly from plasma without full purification, improving turnaround time^[5]^. Future integration of exosome-based diagnostics into sepsis care could enable rapid tests detecting exosomal miRNA combinations (e.g., high miR-155 and low miR-223) to identify hyperinflammatory profiles, prompting early anti-inflammatory therapy, or alert clinicians to rising kidney injury markers before creatinine elevation^[5]^.

*Emerging Therapeutic Strategies Targeting Exosomes in Sepsis* – Beyond diagnostics, exosomes represent both therapeutic targets and tools in sepsis (Table 3). One approach targets the biogenesis or release of harmful exosomes to dampen the cytokine storm and organ injury. Essandoh *et al* demonstrated that administering GW4869, an exosome release inhibitor, to septic mice reduced circulating exosomes, leading to significantly lower inflammation, improved cardiac function, decreased serum TNF-α and IL-6 levels, and higher survival rates^[20]^. This suggests that at least a subset of sepsis pathology is exosome-dependent, and interfering with exosome trafficking might be a viable therapeutic avenue. However, non-specific inhibition of exosomes could also impair beneficial intercellular communications, so precision will be needed (for example, selectively targeting exosomes from certain cell types or carrying certain cargo).

**Table 3:** Exosome-based therapeutic approaches in sepsis. This table lists experimental therapeutic strategies involving exosomes, including cell-derived exosome treatments and engineered exosome interventions, along with their effects in preclinical sepsis models

Therapeutic strategy	Description	Mechanism of action	Observed benefits (preclinical)	Reference
MSC-derived exosome therapy	Infusion of exosomes isolated from mesenchymal stem cells (bone marrow, adipose, etc.)	Exosomes deliver anti-inflammatory miRNAs (e.g., miR-27b, miR-146a) and proteins (TGF-β, angiopoietin-1) to immune cells and injured organs. Shift macrophages from M1 to M2 phenotype; suppress NF-κB signaling; enhance tissue repair	↓ Pro-inflammatory cytokines (IL-6, TNF-α); ↓ lung and kidney injury; improved survival in septic mice	^[26,27]^
Adipose-MSC exosomes (ADSC-Exo)	Exosomes from adipose-derived stem cells	Carry miR-125b-5p, miR-148a-3p, etc. -> activate Nrf2/HO-1 pathway in macrophages (anti-oxidant) and modulate Notch signaling in immune cells	↓ Oxidative stress and cytokine storm; ↓ macrophage IL-27 and lung inflammation; preserved vascular endothelial function (↓ ferroptosis in lung)	^[28,29]^
EPC-derived exosomes (Endothelial Progenitor Cells)	Exosomes from endothelial progenitors	Deliver pro-angiogenic miR-126 and anti-inflammatory lncRNA TUG1 to endothelium and macrophages. Inhibit HMGB1 release, improve microvascular barrier, promote M2 polarization via SIRT1 pathway	↓ Vascular leakage; maintained microcirculation; ↓ sepsis-induced ALI (better oxygenation); ↓ inflammatory macrophages in organs	^[30,31]^
Blockade of exosome release	Pharmacological inhibition (e.g., GW4869) of neutral sphingomyelinase to prevent exosome formation	Reduces the number of circulating exosomes carrying deleterious cargo (inflammatory cytokines, HMGB1, etc.), thereby blunting intercellular propagation of inflammation	In septic mice: ↓ serum IL-1β, IL-6; improved cardiac output; ↑ survival rate vs. untreated sepsis	^[32]^
Decoy exosomes (host-derived)	Stimulation or administration of exosomes that sequester pathogens/toxins	Exosomes with surface receptors (e.g., ADAM10) act as sinks for bacterial toxins, preventing them from binding to host cells. Others carry Ig or complement to opsonize bacteria	In MRSA sepsis model: ↓ hemolysis and cell death; exo-treated mice had improved survival by neutralizing α-toxin. Potential broad toxin neutralization in toxin-mediated sepsis	^[33-35]^
Engineered exosome-mimetic therapy	Synthetic or modified exosomes loaded with therapeutic cargo (e.g., anti-inflammatory miRNAs, siRNAs)	Nanoparticles or donor cells are loaded with specific miRNA combinations that are packaged into exosome-like vesicles. Upon administration, these vesicles enter immune cells and modulate gene expression (e.g., suppressing cytokine production pathways)	Adv. Mater. 2022 study: Biomimetic exosomes with 7 miRNAs drastically ↓ IL-6, TNF levels in septic mice; protected mice and primates from lethal sepsis. Suggests feasibility of tailored exosome therapy to quell cytokine storms	^[33]^
Exosomes as drug delivery vehicles	Use of exosomes to deliver drugs (small molecules, peptides) or gene therapy to target cells in sepsis	Exosomes can be loaded with anti-inflammatory drugs (e.g., curcumin) or siRNA and injected; their natural targeting properties deliver the cargo to macrophages or injured organs, enhancing local drug concentration	e.g., Exosomes loaded with curcumin targeted to inflammatory monocytes increased drug uptake and induced apoptosis of overactive immune cells in septic shock models. Resulted in reduced systemic inflammation compared to free drug. (This approach is under exploration; early results show improved bioavailability and efficacy)	*N/A (conceptual)*

A more refined therapeutic strategy harnesses beneficial exosomes as treatments, with mesenchymal stem cell (MSC)-derived exosomes attracting significant interest. MSCs’ known immunomodulatory and tissue-reparative effects in sepsis are increasingly attributed to their secreted exosomes^[3]^. Multiple animal studies demonstrate that administering purified MSC-derived exosomes reproduces the benefits of cell therapy without the cells themselves. These exosomes carry anti-inflammatory microRNAs and proteins and reduce lung inflammation and injury in septic mice, decreasing alveolar edema and inflammatory cell infiltration^[15]^. Notably, one study found that MSC-derived exosomes delivered miR-27b into macrophages, which suppressed the NF-κB pathway and reduced pro-inflammatory cytokine production^[3]^. This cell-free approach offers the therapeutic benefits of MSCs while avoiding potential complications of direct cell therapy

MSC-derived exosomes demonstrate diverse therapeutic mechanisms in sepsis models. In cecal ligation and puncture (CLP) sepsis, MSC exosome treatment improved survival and organ function by inducing macrophage polarization from pro-inflammatory M1 to anti-inflammatory M2 states via exosomal microRNA cargo^[3]^. Adipose-derived stem cell exosomes showed similar efficacy – Bai *et al* (2020) demonstrated that adipose MSC exosomes delivered miR-148a-3p to macrophages, modulating Notch signaling and alleviating systemic inflammation^[3]^. These exosomes also convey antioxidants, with adipose-derived stem cell (ADSC)-exosomes activating the nuclear factor erythroid 2–related factor 2 (Nrf2)/heme oxygenase-1 (HO-1) antioxidant pathway in immune cells, reducing oxidative damage and cytokine release^[3]^. In sepsis-induced AKI models, bone marrow MSC exosomes improved kidney histology and function through miR-21-5p transfer that inhibited pro-apoptotic pathways in renal cells^[3]^. Research is now moving toward clinical translation, with pilot studies testing MSC-derived exosomes for safety in inflammatory conditions. The appeal of acellular exosome therapy lies in avoiding risks of live cell therapy (uncontrolled replication, embolism) while retaining potent immunomodulatory effects.

Researchers are engineering exosome-mimetic nanovesicles and loading exosomes with specific therapeutic cargo. Li *et al* (2022) created biomimetic immunosuppressive exosomes by exposing tumor cells to lipopolysaccharide (LPS) to generate anti-inflammatory microRNA-enriched exosomes, then formulating synthetic nanoparticles containing seven miRNAs. These artificial exosomes reduced inflammatory cytokines and improved survival in septic mice and non-human primates^[18]^. This approach enables “designer” exosomes tailored to reprogram immune responses – loading them with let-7 family miRNAs for broad inflammatory suppression or siRNAs targeting key mediators like HMGB1 or IL-6.

Another therapeutic concept is using exosomes as drug delivery vehicles in sepsis. Platelet exosomes target endothelium while MSC exosomes accumulate in injured lungs, making them ideal carriers for anti-inflammatory drugs or RNA therapeutics. Preclinical studies have used macrophage-derived exosomes loaded with anti-miR-155 to successfully reduce inflammatory damage in lungs and liver of septic mice^[2,4]^.

Additionally, therapies aim to augment beneficial endogenous exosomes. Encouraging release of decoy exosomes that neutralize toxins, like ADAM10-bearing exosomes against bacterial α-toxin, could benefit septic patients with toxin-producing infections^[11]^. Treatments stimulating autophagy-related protein 16-1 (ATG16L1)-mediated exosome release might enhance toxin scavenging, while enhancing neutrophil release of protease-rich exosomes could help clear fibrin clots in DIC^[23]^. These approaches illustrate the range of exosome-targeted interventions from suppressing harmful vesicles to supplementing protective ones.

The therapeutic implications of exosome research in sepsis are far-reaching, with exosomes representing both targets to inhibit pathological communication and tools as therapy carriers. Preclinical evidence strongly supports exosome-based therapies, particularly MSC-derived exosomes, for attenuating inflammation, oxidative stress, and apoptosis in sepsis^[3]^. Engineered exosomes tackle challenges like cytokine storm by delivering concentrated immunomodulatory signals^[18]^. While not yet in routine clinical practice, these approaches are progressing toward clinical trials, though key challenges including scalable production, dosing, and ensuring exosome homogeneity remain. Given their ability to orchestrate inter-cell communication, exosomes are poised to become both biomarkers and “bio-medicines” in the fight against sepsis.

## Major unresolved debates and conflicting findings in sepsis exosome research

1. **Divergent Functional Roles (Protective vs. Harmful**): The central debate in sepsis exosome research revolves around their functional role – whether exosomes act as protective or harmful agents. Studies demonstrate that sepsis significantly alters circulating exosome levels and cargo composition, but their effects appear dualistic and context-dependent^[26]^. While some evidence indicates exosomes exacerbate tissue damage by propagating inflammatory signals, other data suggest they carry anti-inflammatory or tissue-repair factors that improve outcomes^[27]^. This dichotomy often depends on the cellular source and disease phase, with exosomes released during the initial hyperinflammatory phase potentially amplifying organ damage, whereas those produced in later immunosuppressive phases might convey protective signals.
2. **Source Heterogeneity**: Another major controversy centers on identifying which exosome-producing cells drive sepsis pathophysiology. Virtually all cell types, including immune cells, platelets, endothelial cells, and erythrocytes, shed exosomes during sepsis, making it unclear which source is most critical. Different studies focus on different exosome subpopulations, leading to inconsistent conclusions. For instance, macrophage-derived exosomes have shown both pro-inflammatory, tissue-damaging effects^[28]^ and anti-inflammatory or antibacterial functions^[29]^ in different experimental contexts. This heterogeneity of exosome populations, varying greatly by cell origin, cargo, and timing, makes it challenging to establish universal mechanisms.
3. **Biomarker Inconsistencies**: The biomarker potential of circulating exosomes remains highly controversial due to inconsistent findings across studies. Despite dozens of candidate exosomal microRNAs, lncRNAs, and proteins being identified as potential sepsis biomarkers, there is little consensus on which are truly reliable^[5]^. For example, exosomal microRNAs like miR-150-5p, miR-30d-5p, and miR-181a show different patterns across studies – some report significant alterations correlating with organ injury, while others fail to validate these findings^[33]^. This lack of reproducibility means that many promising exosomal biomarkers in single studies lack clinical validation across larger, diverse patient groups, and no exosome-based biomarker has yet achieved the consistency of traditional markers like procalcitonin. Examples of reported exosomal miRNA biomarkers in sepsis with inconsistent results across studies including miR-21-5p^[30,31]^, miR-181a/miR-23b^[32,34]^, and miR-1298-5p^[31]^.
4. **Methodological Discrepancies**: Methodological discrepancies significantly contribute to these conflicting findings. Different laboratories employ varying protocols for exosome isolation (ultracentrifugation, filtration, precipitation kits), quantification, and cargo analysis, with no universally accepted gold standard method^[35]^. This lack of standardization leads to variable results. While efforts to improve standardization through guidelines from organizations like the International Society for Extracellular Vesicles are underway, significant challenges remain in achieving uniform quality controls necessary for drawing firm conclusions across laboratories.

## Emerging technologies to overcome current limitations in sepsis-exosome studies

Innovative tools and methodologies are now being explored to resolve the above challenges and deepen our understanding of exosomes in sepsis. Several promising technologies from the past few years could help overcome current limitations:

1. **Single-Exosome Profiling and High-Resolution Analysis**: Advanced single-particle analysis techniques are revolutionizing exosome research by revealing heterogeneity in vesicle populations previously masked in bulk studies. Technologies like proximity barcoding assays^[36]^, nano-flow cytometry^[37]^, interferometric imaging^[38]^, and ExoView chips^[39]^ enable researchers to profile thousands of individual exosomes, identifying distinct subpopulations with specific molecular signatures. This approach can resolve conflicting findings in sepsis research by distinguishing pro-inflammatory from anti-inflammatory vesicle subsets and detecting rare but informative exosome populations that may serve as better biomarkers. By identifying specific “harmful” versus “protective” exosome types on a per-particle basis, these tools provide unprecedented resolution for understanding mechanistic effects and improving biomarker discovery.
2. **CRISPR-Engineered Exosomes**: CRISPR technology has emerged as a powerful tool for modifying exosome content and function. Researchers can engineer parent cells to produce exosomes with desired traits by knocking out harmful components or introducing therapeutic cargo. Additionally, exosomes can deliver CRISPR/Cas9 machinery to target cells for gene editing with high specificity and low off-target effects^[40,41]^. In sepsis research, CRISPR-engineered exosomes serve dual purposes: as research tools to test hypotheses by selectively disabling suspected harmful cargo or loading protective factors, and as potential therapeutics to modulate immune responses or target pathogens^[42]^. This technology enables precise manipulation of exosomal content, allowing rigorous testing of their role in sepsis pathophysiology.
3. **Microfluidics and Nanosensor Technologies**: Microfluidic devices and nanosensors are addressing the technical challenges of exosome isolation and detection^[43,44]^. Lab-on-a-chip systems with nanoscale channels or antibody-coated surfaces can rapidly purify exosomes from minimal blood samples with high specificity, eliminating contaminants common in ultracentrifugation. Novel biosensors including nano-plasmonic sensors^[45]^, surface plasmon resonance imaging^[46]^, total internal reflection fluorescence microscopy^[47]^, and nanopore sensors^[48]^ enable accurate single-vesicle quantification and characterization. These technologies reduce sample loss, standardize analysis across laboratories, and enable real-time exosome profiling in clinical settings. By improving isolation purity and detection sensitivity, these innovations directly address the reproducibility issues plaguing current exosome research.
4. **Other Innovative Approaches**: Additional emerging tools include advanced omics techniques combined with machine learning to identify robust exosomal biomarker signatures from complex datasets. Exosome mimetics offer standardized synthetic alternatives to natural exosomes for research and therapeutic applications. Improved in vivo imaging using fluorescent or radiolabeled tracers allows tracking of exosome distribution and fate in septic models, providing crucial insights into their functional impact. Together with single-exosome analysis, CRISPR engineering, and microfluidic sensors, these expanding technologies are paving the way for more definitive answers about exosomes’ role in sepsis, promising to resolve current controversies and enhance data reliability.

## Clinical translation challenges

Translating exosome research into clinical applications (be it a diagnostic test or a therapy for sepsis) faces several critical hurdles. Despite exciting progress, no exosome-based therapy or diagnostic has yet achieved regulatory approval for sepsis, largely due to the following challenges:

1. **Standardization and Quality Control**: The lack of standardized protocols for exosome production and characterization represents a fundamental barrier to clinical translation. Exosomes are inherently heterogeneous, and variations in isolation methods, purification levels, and storage conditions dramatically affect their composition and function. For regulatory approval, any exosome-based diagnostic or therapeutic must be produced under Good Manufacturing Practice with strict quality control measures including defined release criteria for purity, potency, and identity. Current research suffers from inconsistent methodologies across laboratories, making it difficult to define reproducible products. While consensus guidelines like Minimal Information for Studies of Extracellular Vesicles (MISEV) standards^[49]^ have emerged to improve reproducibility, the field still lacks widely adopted standard operating procedures. This means sepsis exosome biomarker assays must undergo validation with standardized sample processing, and therapeutic products require clear protocols ensuring consistent properties across batches.
2. **Manufacturing and Scale-Up**: Unlike small-molecule drugs, exosomes present complex biological products that are challenging to mass-produce. Current research methods yield small amounts of exosomal material through lengthy ultracentrifugation processes, insufficient for clinical applications. Scaling production to generate clinically relevant therapeutic doses or process large numbers of diagnostic samples remains a major hurdle. While companies explore bioreactor systems and cell-free production methods, maintaining quality at scale proves difficult due to batch variability, contamination risks, and decreased bioactivity. Purity presents another manufacturing challenge, requiring advanced techniques like tangential flow filtration and size-exclusion chromatography to replace traditional ultracentrifugation. Producing clinical-grade exosomes with consistent cargo loading and characteristics at gram-scale quantities remains largely experimental, necessitating development of robust bioprocessing methods that include comprehensive lot release testing similar to biologic drugs.
3. **Safety and Immunogenicity**: Rigorous safety evaluation is essential for any exosome-based therapy, particularly in sepsis patients with dysregulated immune systems. While exosomes are naturally occurring, large doses or allogeneic sources could provoke immune reactions. Safety concerns include potential unwanted components like pro-inflammatory cytokines or nucleic acids that could trigger immune activation or exacerbate coagulation abnormalities in septic patients. Biodistribution and clearance patterns must be carefully monitored, as exosomes typically accumulate in liver, spleen, or lungs – organs often compromised in sepsis. Long-term safety questions remain regarding potential immune system alterations or genomic effects, especially with engineered exosomes carrying RNAs or gene editors. While early clinical trials in other fields report no serious adverse events, the fragile state of septic patients demands extensive toxicity studies, immunogenicity assays, and in vivo tracking to ensure biocompatibility and define safe dosage ranges.
4. **Regulatory and Approval Hurdles**: The regulatory pathway for exosome-based products remains poorly defined, as exosomes occupy a gray zone between traditional biologics and cell therapies. Regulatory agencies like the FDA currently classify exosomes as biological drug products, requiring full Investigational New Drug applications and comprehensive clinical trials. No exosome-based therapy or diagnostic has achieved FDA approval as of 2025. Key regulatory challenges include demonstrating consistent Chemistry, Manufacturing, and Controls (CMC), defining mechanisms of action and potency assays despite exosomes’ complex cargo, and establishing clear efficacy endpoints for sepsis trials. Diagnostic applications face regulation as in vitro diagnostic devices, requiring extensive clinical validation to prove accuracy, specificity, and added value over existing tests. The regulatory landscape demands satisfaction of stringent requirements for quality, consistency, safety, and efficacy, necessitating collaboration between academia, industry, and regulators to establish clear guidelines and standards for exosome products in sepsis applications.

## Conclusion

Sepsis profoundly influences circulating exosomes, transforming them into both messengers of dysregulated immunity and promising clinical tools. The septic inflammatory environment drives increased release of exosomes loaded with inflammatory cytokines, coagulation factors, and damage signals that disseminate injury to distant organs. Simultaneously, compensatory mechanisms pack immunosuppressive and pro-resolving cargo into exosomes, contributing to the complex immune paralysis seen in later sepsis. These small vesicles mirror the immunometabolic state of the host, making them valuable “fingerprints” that can be detected and analyzed. Emerging evidence suggests that exosomal biomarkers – proteins like HMGB1, miRNAs such as miR-122, and even lncRNAs/circRNAs – could enhance our ability to diagnose sepsis early, monitor organ damage, and stratify patients for tailored therapies.

**Figure 1.**
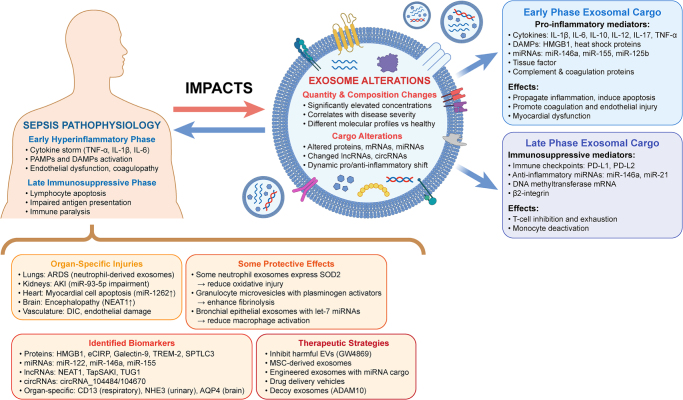
This diagram illustrates how sepsis pathophysiology impacts exosome characteristics and how exosomes contribute to sepsis progression. The septic process induces both quantity and compositional changes in circulating exosomes, with distinct cargo profiles in early pro-inflammatory and late immunosuppressive phases. Exosomal cargo includes cytokines, DAMPs, miRNAs, and coagulation factors that propagate inflammation and cause organ-specific injuries. While most exosomes exacerbate sepsis, some contain protective factors that reduce oxidative injury and inflammation. The diagram also highlights exosomal biomarkers and emerging therapeutic strategies targeting harmful exosomes or delivering protective cargo.

The burgeoning field of exosome research in sepsis offers exciting therapeutic possibilities. Inhibiting harmful exosome release has improved outcomes in animal models, while administering beneficial exosomes (particularly from stem cells) has demonstrated multi-organ protective effects. Engineered exosome-mimetics carrying specific anti-inflammatory cargo have shown remarkable ability to calm cytokine storms and enhance survival in preclinical studies. Despite challenges in standardization and production, exosome-based therapeutics represent a promising frontier in sepsis management. Figure 1 provides a diagrammatic illustration of the reciprocal interaction between sepsis and exosomes. By understanding how exosomes both reflect and participate in sepsis pathophysiology, researchers and clinicians are developing novel diagnostic and treatment approaches with the potential to significantly improve outcomes in this devastating condition with persistently high mortality rates.

## Data Availability

Data sharing not applicable – no new data generated.

## Announcement of AI assistance

In the preparation of this narrative review, the authors utilized a generative AI tool to assist with writing and editing tasks, in accordance with the Transparency in the Reporting of Artificial Intelligence (TITAN 2025) guidelines^[50]^. Specifically, OpenAI’s ChatGPT (GPT-4o model) was employed for language polishing, formatting consistency, paragraph restructuring, citation cross-checking, and summary refinement. The AI provided support by correcting grammar and spelling, ensuring abbreviations were used consistently, rephrasing sentences for clarity, detecting duplicate references in the bibliography, and refining the summary of certain sections. Importantly, the AI was not used to generate any original ideas or content de novo; it only analyzed and suggested improvements for text written by the authors. All AI assistance was obtained via manually entered prompts using the official ChatGPT interface, with no plug-ins, scripts, or local AI deployments involved. The tool was used in its standard cloud-based configuration. AI-proposed changes were fact-checked against source literature and the authors’ own knowledge to ensure no alteration of intended meaning or introduction of errors. Any AI-generated phrasing that was unclear, incorrect, or inconsistent with the manuscript’s academic tone was edited or discarded. The authors retained full control over the writing process throughout and take full responsibility for the integrity and scientific accuracy of the final manuscript. The authors further disclose that they have no financial ties or conflicts of interest related to the AI tool used.
